# Glycolysis drives STING signaling to promote M1-macrophage polarization and aggravate liver fibrosis

**DOI:** 10.7150/ijbs.115073

**Published:** 2025-10-01

**Authors:** Ping Chen, Zijing Zhu, Wenjie Chen, Zhiyu Xiong, Kan Shu, Muhua Sun, Yingan Jiang, Lanjuan Li

**Affiliations:** 1 Department of Infectious Diseases, Renmin Hospital of Wuhan University, Wuhan, 430060, China.; 2 Department of Urology, The Second Affiliated Hospital, Jiangxi Medical College, Nanchang University, Nanchang, 330006, Jiangxi, China; 3 Division of Nephrology, Renmin Hospital of Wuhan University, Wuhan, 430060, China.; 4 State Key Laboratory for Diagnosis and Treatment of Infectious Diseases, National Clinical Research Center for Infectious Diseases, Collaborative Innovation Center for Diagnosis and Treatment of Infectious Diseases, The First Affiliated Hospital, College of Medicine, Zhejiang University, Hangzhou, 310003, China.

**Keywords:** glycolysis, innate immune response, liver fibrosis, macrophage

## Abstract

Glycolysis activation plays a critical role in sustaining the proinflammatory phenotype of macrophages, which is key to initiating and advancing liver fibrosis. However, the underlying mechanisms that trigger glycolytic activation and their contribution to inflammation remain poorly understood. In this study, we showed that inhibiting glycolysis markedly suppresses macrophage M1 polarization and alleviates liver inflammation and fibrosis, whereas enhancing glycolysis in hepatic macrophages produces the opposite effect. Additionally, our results demonstrated that glycolytic flux is necessary for activation of the STING/TBK1/IRF3 pathway. Moreover, STING activation was found to reciprocally stimulate glycolysis in macrophages. Mechanistically, we found that ATP generated through glycolysis promotes STING pathway activation and enhances the interferon-dependent immune response. Moreover, activation of IRF3, a downstream transcription factor of STING, upregulates HIF-1α transcription, further driving glycolysis. These findings uncover novel mechanistic links between STING signaling and glycolytic metabolism, emphasizing their coordinated role in promoting macrophage M1 polarization. Together, our data suggest that targeting the interaction between metabolic reprogramming and immune signaling offers an effective therapeutic approach for treating liver fibrosis and cirrhosis.

## Introduction

The increasing prevalence of chronic liver diseases presents a major global health challenge and contributes significantly to the worldwide economic burden^1^. Liver fibrosis (LF) is a critical intermediate stage in the progression of numerous chronic liver disorders toward cirrhosis, hepatocellular carcinoma, and eventual liver failure. Evidence suggests that persistent, unresolved hepatic inflammation—primarily triggered by recurrent or chronic hepatocyte injury from diverse etiological factors—is central to the development of LF^2^, ^3^. However, due to the complex and multifaceted pathogenesis of LF, no clinically approved antifibrotic therapies are currently available.

Macrophages, as key players in innate immunity, are deeply implicated in the onset and progression of LF^4^. Upon stimulation by pathogen-associated molecular patterns or damage-associated molecular patterns, liver-resident macrophages (Kupffer cells) secrete a cascade of proinflammatory cytokines and chemokines that drive immune cell recruitment and exacerbate hepatic inflammation^5^. In addition, transforming growth factor-β released by activated Kupffer cells stimulates hepatic stellate cells (HSCs), promoting excessive collagen synthesis and fibrotic remodeling^6^. Importantly, macrophages undergo metabolic reprogramming in response to proinflammatory signals, characterized by a metabolic shift from oxidative phosphorylation to aerobic glycolysis. This glycolytic reprogramming enables rapid ATP production, supporting the heightened biosynthetic and secretory demands of M1-type macrophages^7^. Given that the liver is the principal site of endogenous glucose production and glucose homeostasis^8^, it is particularly vulnerable to metabolic dysregulation. Nonetheless, the precise mechanisms by which augmented macrophage glycolysis contributes to hepatic inflammation remain insufficiently defined.

The stimulator of interferon genes (STING) is a central mediator of innate immune responses and plays a vital role in maintaining hepatic immune balance by activating the NF-κB and IRF3 pathways, which in turn drive interferon (IFN) and cytokine production^9^. Recent studies have increasingly highlighted the involvement of STING signaling in various liver pathologies^10^, ^11^. Aberrant STING activation—induced by mitochondrial DNA leakage—has been reported in nonalcoholic fatty liver disease (NAFLD), acute liver injury, and autoimmune hepatitis^12^, ^13^. Intriguingly, recent evidence suggests that glycolysis, independent of mitochondrial DNA leakage, also triggers STING pathway activation^14^. Despite these findings, the reciprocal interactions between enhanced glycolysis and STING signaling in macrophages during LF remain poorly defined.

This study aims to elucidate the crostalk between macrophage glycolysis and STING signaling in the context of LF by focusing on the metabolic adaptability of macrophages. These insights may offer a conceptual framework for the development of targeted therapies to alleviate LF.

## Materials and Methods

### Construction of myeloid-specific STING-knockout mice

We generated myeloid-specific STING knockout (STING^fl/fl^/Lyz2-Cre, STING^M-KO^) mice by crossing C57BL/6J background homozygous floxed STING (STING^fl/fl^) mice (stock#031670; Jackson Lab) with Lyz2-Cre mice (C57BL/6J, stock# C001003; Cyagen), while STING^fl/fl^ mice served as controls. Genotyping for STING^M-KO^ mice was performed when the mice reached the age of 2 weeks using the following primers for PCR (sequence 5'-3'):

Cre-Forward: CTTGGGCTGCCAGAATTTCTC; Cre-Reverse: CCCAGAAATGCCAGATTACG; LoxP-Forward: TTT TCA TCT GCC TTC CAG GT; LoxP-Reverse: GCG CAC ACA CAC TAA AAA CTG.

### Construction of a hepatic macrophage HK2-overexpression murine model

To generate a hepatic macrophage (MФs) HK2-overexpression murine model, we used AAV9 viral vectors to deliver the MФ-AAV9-F4/80-HK2 (F4/80) promoter, which drives the HK2 expression in hepatic MФs, into C57BL/6J mice via intrahepatic injection. The recombinant AAV9 was produced in HEK293T cells using pAAV-RC and pHelper, as described previously^15^, ^16^. The AAV9 viral vectors encoded *HK2* under the control of the macrophage AAV9-F4/80-HK2 promoter. In addition, an empty vector under the regulation of the AAV9-F4/80 promoter (AAV9-F4/80-NC) was constructed concurrently. The viral titers for AAV9-F4/80-HK2 and AAV9-F4/80-NC were both 1.6 × 10¹² vector genomes (vg)/mL. Subsequently, 6 male C57BL/6J mice (age 6 weeks) were administered a 200 μL injection of either AAV9-F4/80-HK2 or AAV9-F4/80-NC vector, and the overexpression efficiency was confirmed 3 weeks after the injection.

### Liver fibrosis model and treatment

The animal experiments conducted in this study received approval from the Ethics Committee of Wuhan University and were conducted as per the Guide for the Care and Use of Laboratory Animals.

Wild-type (WT) male C57BL/6 mice (weight: 18-23 g, age: 6-8 weeks) were obtained from GemPharmatech Co., Ltd. (Jiangsu, China). All mice were housed under a standard pathogen-free laboratory environment with unrestricted access to water and chow diet, and were then randomly assigned to each group. The formal experiment was conducted after 7 days of acclimatization. All animals included in the study were subjected to the experimental procedures, and no animals were excluded from the analysis.

WT, STING^fl/fl^, and STING^M-KO^ mice (n = 6 in each group) were injected intraperitoneally (i.p.) with either a vehicle (normal saline) or 1% thioacetamide (TAA; Sigma, 163678) at a dose of 150 mg/kg body weight. The TAA treatment was administered from the third week for 12 consecutive weeks so as to induce liver fibrosis and assess the role of STING in this process.

To examine the effect of glycolysis inhibition on established liver fibrosis, the experimental mice were injected i.p. with 2-deoxy-D-glucose (2-DG) at a dose of 1 g/kg/mouse every 3 days during the injection of TAA, in accordance with a previously described method^17^, ^18^. To examine the role of macrophage HK2 overexpression in liver fibrosis, the mice were first pretreated with AAV9-F4/80-HK2 and AAV9-F4/80-NC vector injection and then with 1% TAA (150 mg/kg, i.p.) from the third week for 12 weeks. After 2 days of the final treatment with 2-DG, the mice were euthanized and sacrificed, and their livers and serum samples were collected for subsequent analyses.

### Hepatic histopathological examination and serum biochemistry

After fixing the liver tissues with 4% paraformaldehyde (PFA) and embedding them in paraffin, 5-μm-thick slices were cut and stained with hematoxylin and eosin (H&E), Masson's trichrome, and Sirius Red for liver histopathological analyses.

The serum levels of aspartate aminotransferase (AST) and alanine aminotransferase (ALT) were assessed using AST and ALT kits (ELK1778 and ELK1921, respectively; ELK Biotechnology) as per the manufacturer's protocol.

### Bone marrow-derived macrophages (BMDMs) isolation and treatment

The bone marrow cells were isolated from STING^M/KO^, STING^fl/fl^, and WT mice (age: 6 weeks) and induced into macrophages (BMDMs). Briefly, after their euthanasia, the tibias and femurs of the mice were harvested and immersed in 75% ethanol for 5 min, and the bone marrow cells were flushed out using pre-cold PBS buffer. After centrifugation (1300 r.p.m. for 5 min at 4 °C), the pellets were resuspended in red blood cell lysis buffer (BL503A; Biosharp) for 5 min. The cells were then cultured in complete DMEM (Hyclone, USA) medium supplemented with 20 ng/mL M-CSF (315-02; PeproTech). The culture medium was refreshed on the third day, and the bone marrow cells were differentiated into macrophages on the 7^th^ day at 37 °C in a 5% CO_2_ incubator.

### *In vitro* experimental design

The following experimental groups were established: Normal control (NC): BMDMs from WT, STING^fl/fl^, and STING^M/KO^ mice were stored in complete medium (DMEM medium supplemented with 10% FBS and 1% penicillin‒streptomycin); (2) Lipopolysaccharide (LPS) group: for classical macrophage activation, BMDMs from WT, STING^fl/fl^, and STING^M/KO^ mice were stimulated with LPS (L2880; Sigma) 100 ng/mL for 12 h; (3) 2-DG group: BMDMs from WT mice were cultured in a complete medium containing 2-DG (5 mM); (4) LPS+2-DG group: BMDMs from WT mice were pre-treated with 5 mM 2-DG for 12 h and then challenged with LPS (100 ng/mL) for another 12 h. (5) si-IRF3 group: BMDMs were pre-transfected with small-interfering RNA (siRNA) targeting IRF3 or a negative control (si-NC) using Lipo3000 reagent (Invitrogen, USA) for 48 h, followed by LPS stimulation for 12 h.

siRNA fragments targeting IRF3 were synthesized by Sangon Biotech (Wuhan, China), and the sequence was as follows (5'-3'): sense: GGUUCAGGAUCCCGUGGAA'; antisense: UUCCACGGGAUCCUGAACC'.

### Supplementation with ATP

The protocol was conducted in accordance with previously described methods^14^, ^19^. Briefly, BMDMs were treated with SLO (SAE0089, 800 ng/mL; Sigma) for 1 h, followed by 5 mM ATP (MCE, HY-B2176) for an additional 2 h. After treatment, BMDMs were exposed to LPS for 12 h. Moreover, to investigate the effects of glycolytic inhibition *in vitro*, BMDMs were pretreated with 2-DG before proceeding with the abovementioned experimental procedures. Finally, BMDMs were collected for WB analysis and ATP detection.

### Lactate production assay

A Lactate Assay Kit (Cat# ab65331, Abcam) was applied to measure the L-lactate production in BMDMs and in mouse liver tissues. Then, 10 mg of the tissues or 2 x 10^6^ macrophages were homogenized in 200 μL lactate assay buffer and centrifuged for 5 min at 12,800 rpm at 4°C. Then, the supernatants were collected into a clean tube, and lactate substrate mix and lactate enzyme mix were added into the tube to prepare the reaction mixture, which was then incubated for 30 min at the room temperature (RT), followed by determination of the lactate production by a microplate reader at OD 450 nm.

### Flow cytometry analysis

To analyze macrophage polarization *in vitro*, BMDMs from among different groups were incubated with FITC-F4/80 and APC-Cy7-CD86 antibodies (123107 and 105029, respectively, Biolegend) at 4°C in the dark for 1 h, after washing and resuspending with cell-staining buffer, and the macrophages were subsequently analyzed by flow cytometry (Beckman, USA). All experiments were performed independently in triplicate, and the data were processed with FlowJo software.

### Immunofluorescence (IF) and immunohistochemistry (IHC) staining

All primary and secondary antibodies used for IF and IHC are detailed in Supplementary Table 1. BMDM cell slides and 5-μm-thick liver sections were fixed with 4% PFA for 17 min and then blocked in 5% BSA buffer for 40 min, followed by incubation with primary antibodies (F4/80, CD86, CD206, HK2, p-STING, p-IRF3, and HIF-1α) overnight at 4 °C. The sections or slides were labeled with Alexa Fluor 647, FITC 488-conjugated, or Cy3-labeled secondary antibodies for 1 h at RT. In addition, DAPI (D1306; Invitrogen) was used for nuclear counterstaining. Then, the IF images were captured by a fluorescence microscope (Olympus, Japan) and analyzed by Image J software.

For IHC staining, paraffin-embedded liver slices were processed for antigen retrieval after dewaxing and rehydration. Next, the slices were blocked with 5% normal goat serum (ab7481; Abcam) at RT for 1 h, and endogenous peroxidases were quenched with 3% H_2_O_2_. The slices were incubated overnight at 4°C with primary antibodies against -α-SMA, Col1a1, HK2, and Ly6g. After washing with TBST, horseradish peroxidase (HRP)-conjugated secondary antibodies diluted at 1:3000 were added onto the slices. Finally, the slides were developed with DAB substrate kit (P0203; Beyotime) and then counterstained with hematoxylin. IHC images were photographed using the Olympus microscope. The positively stained areas were quantified with ImageJ software.

### Western blotting (WB) analysis

RIPA lysis buffer (Servicebio, China) was used for extracting proteins from the mice liver tissues or cultured BMDMs. The protein concentration was determined by BCA assay, and equal amounts were separated by SDS-PAGE, transferred onto PVDF membranes, and probed with primary antibodies overnight. The membranes were then incubated with HRP-conjugated secondary anti-mouse or anti-rabbit antibodies for 1 h. Signal detection was performed using the QuickChemi 5200 system, and the band intensities were quantified with ImageJ. All antibodies used in WB are listed in Supplementary Table 1.

### Quantitative real‑time PCR (RT-qPCR)

RNA was extracted from BMDMs and liver tissues using Trizol reagent (Invitrogen). After determining the concentration, 3 μg RNA was reverse-transcribed and amplified into cDNA by using the cDNA Synthesis Kit (RK20433; Abclonal). Subsequently, qPCR was conducted using the universal SYBR Green qPCR mix (RK21203; Abclonal). The primer sequences used for RT-qPCR are detailed in Supplementary Table 2. The relative mRNA expression of the target genes was quantified by the 2^-ΔΔCT^ method, normalized to β-actin, and then analyzed with the Roche LightCycler^®^ 480 system.

### Enzyme-linked immunosorbent assay (ELISA)

The BMDMs culture medium supernatants and mouse serum were collected for cytokine analysis. ELISA kits (Elabscience, China) were used to quantify the levels of TNF-α, IFN-β, IL-6, IL-1β, and IL-10 (Supplementary Table 3), and the assay was conducted following the manufacturer's protocols.

### Extracellular acidification rate (ECAR) analysis

The ECAR of BMDMs was measured using the Seahorse XF Glycolytic Rate Assay Kit (103020-100; Agilent) in accordance with the manufacturer's instructions. Briefly, the day before the experiment, the indicated BMDMs were plated at XF24 cell culture microplates (Agilent). The next day, the medium was exchanged with XF DMEM medium (103015-100; Agilent) and cultured in a CO_2_-free incubator at 37 °C for 1 h. Subsequently, the following substances were added at the designated timepoints: 10 mM glucose (glycolysis induction), 1 μM oligomycin (to maximize glycolysis), and 50 mM 2-DG (to inhibit glycolysis). The real-time ECAR data were automatically recorded by the XFe24 Extracellular Flux Analyzer (Agilent).

### Dual-luciferase reporter assay

BMDMs were seeded into six-well plates. The potential binding site of IRF3 on the HIF-1α promoter was predicted using the Animal Transcription Factors Database (AnimalTFDB4.0, https://guolab.wchscu.cn/AnimalTFDB4/#/). Dual-luciferase reporter assay was performed to identify the targeting relationship between IRF3 and the HIF-1α promoter. Briefly, WT or mutant HIF-1α-promoter sequences (WT or Mut, respectively) were constructed into the pGL3 vector, whereas IRF3 was constructed into the pEnCMV-3xflag vector (Qinda Biol Co., LTD., China). Subsequently, the firefly luciferase plasmids pGL3-HIF-1α-WT and pGL3-HIF-1α-Mut were co-transfected with pEnCMV-3xflag-NC (WT-IRF3), pEnCMV-3xflag-IRF3 (used for IRF3 overexpression *in vitro*, OE-IRF3), and pRL-TK renilla luciferase vector (internal reference) into BMDMs. After 48 h, a Dual-Luciferase Reporter Assay Kit (DL101-01; Vazyme) was applied to measure the firefly and renilla luciferase activities according to the manufacturer's protocol. The luminescence signal was recorded by using a microplate reader (EnSight; PerkinElmer).

### Molecular docking analysis

To explore the interaction between IRF3 and HIF-1α, molecular docking analysis was performed. The crystal structure of mouse-IRF3 protein was acquired from the UniProt database (Uniprot ID: P70671), and the HIF-1α-promoter sequence was obtained from the NCBI database. According to the HIF-1α-promoter sequence, AnimalTFDB4.0 was used to predict the binding sites of IRF3 on the HIF-1α-promoter region. HDOCKlite v1.1 was employed to execute protein-nucleic acid docking analysis between HIF-1α and IRF3 proteins, and the result was visualized using PyMOL software. The two key indicators used for evaluating the docking results included: first, the docking score, where a greater negative value signifies a higher likelihood of an optimal binding model, and, second, the confidence score, when the value >0.7 indicates a high probability of the binding relationship.

### Targeted metabolomics analysis

Targeted metabolite profiling of BMDMs was performed using LC/MS-MS and conducted by Metware Biotechnology Co., Ltd. (Wuhan, China). Briefly, BMDMs isolated from STING^M/KO^ and STING^fl/fl^ were stimulated without or with LPS, and the cells were collected and stored at -80 °C. Each group contained 4 duplicates. Next, the samples were dispersed with 800 μL of cold methanol/acetonitrile (1:1) and subsequently ultrasonicated for 20 min in an ice bath. Subsequently, the samples were centrifuged for 20 min (14000 x*g*, 4 °C) and the supernatants were harvested. Finally, the supernatants were analyzed by QTRAP™ 6500 LC-MS/MS System. Data were analyzed by Cluster 3.0 and the Java Treeview software. Differentiated metabolites were identified using Fold Change > 1.5, padj < 0.05 as the criteria.

### Statistical Analyses

All statistical analyses were conducted using Student's *t*-test or one-way analysis of variance (ANOVA), followed by Turkey's test for comparison among groups. Data were expressed as the mean ±SEM and were analyzed by GraphPad Prism 9.5 software (GraphPad, USA). *p* < 0.05 indicated statistical significance.

## Results

### Increased macrophage glycolysis in LF

**Figure 1:**
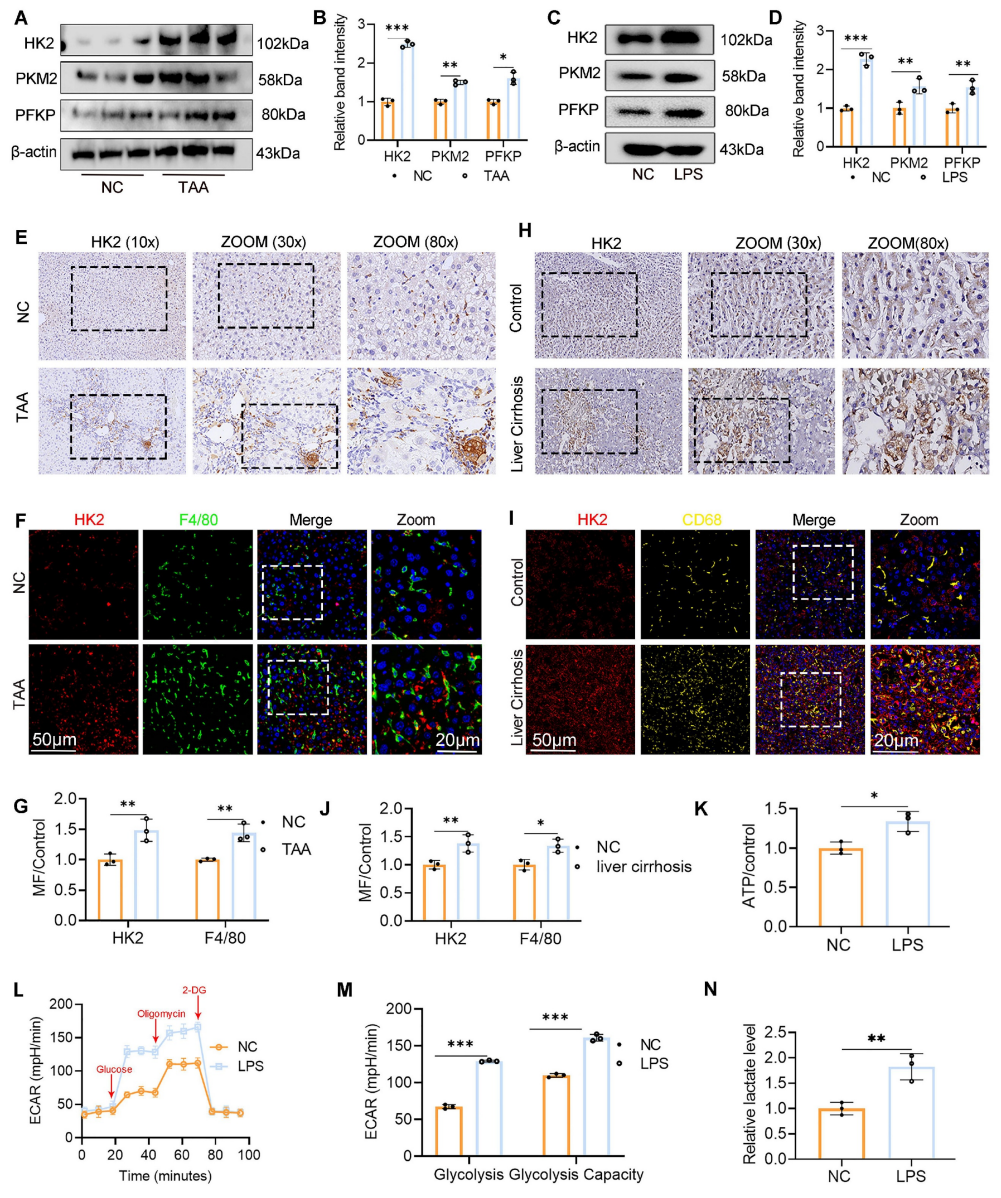
** Increased macrophage glycolysis was observed in liver fibrosis (LF). (A-B)** Protein expression levels of HK2, PKM2 and PFKP in liver tissues from normal control and TAA-injected mice. **(C-D)** Western blot analysis of HK2, PKM2 and PFKP protein expression in BMDMs. **(E-G)** Immunohistochemical (IHC) staining of HK2 and immunofluorescence (IF) staining of F4/80 (green) and HK2(red) in liver sections of mice, respectively. Magnification: 10x; zoom at 30x and 80x. Scale bar = 50 μm, 20 μm (zoom). **(H-J)** IHC staining of HK2 and representative IF images and quantifications of F4/80 (green) and HK2(red) and in human liver sections, respectively. Magnification: 10x; zoom at 30x and 80x. Scale bar = 50 μm, 20 μm (zoom). **(K)** Relative ATP content in BMDMs treated with or without LPS. **(L-M)** Extracellular acidification rate (ECAR) analysis in cultured BMDMs. **(N)** Relative lactate levels in cultured BMDMs. *p < 0.05, **p < 0.01, ***p < 0.001.

To investigate the association between macrophage glycolysis and LF, we established a TAA-induced LF model in WT C57BL/6J mice. Additionally, BMDMs were generated from WT mice and polarized to the M1 phenotype by LPS stimulation. WB analysis was performed to assess the expression of key glycolytic rate-limiting enzymes, including hexokinase 2 (HK2), pyruvate kinase M2, and phosphofructokinase platelet type, both *in vivo* and *in vitro*. All three enzymes were significantly upregulated in liver tissues from TAA-treated mice and in LPS-stimulated BMDMs compared with the NC group. Notably, HK2 exhibited the most pronounced increase in expression (Fig. 1A-D). IHC staining further confirmed the elevated HK2 expression in the livers of TAA-treated mice (Fig. 1E). We also evaluated HK2 expression in liver samples from patients with cirrhosis and NCs. IHC analysis revealed significantly greater HK2 positivity in liver sections from patients with cirrhosis than in those from NCs (Fig. 1H). Additionally, double IF staining for F4/80 and HK2 demonstrated elevated HK2 expression specifically in macrophages within both human and murine fibrotic liver tissues (Figs. 1F-G, 1I-J).

To assess glycolytic flux in macrophages, ECAR was measured. The nonglycolytic acidification rate remained comparable between LPS-stimulated and control BMDMs. However, following glucose and oligomycin administration, the glycolytic acidification rate significantly increased in LPS-stimulated BMDMs, indicating enhanced glycolytic activity in M1-polarized macrophages upon LPS exposure (Figs. 1L-M). Furthermore, ATP and lactate levels were measured to assess glucose metabolism, and both were found to be elevated in activated BMDMs (Figs. 1K, N). Collectively, these findings demonstrate that increased macrophage glycolysis is a defining metabolic feature in LF.

### 2-DG treatment ameliorates TAA-induced mice LF

**Figure 2:**
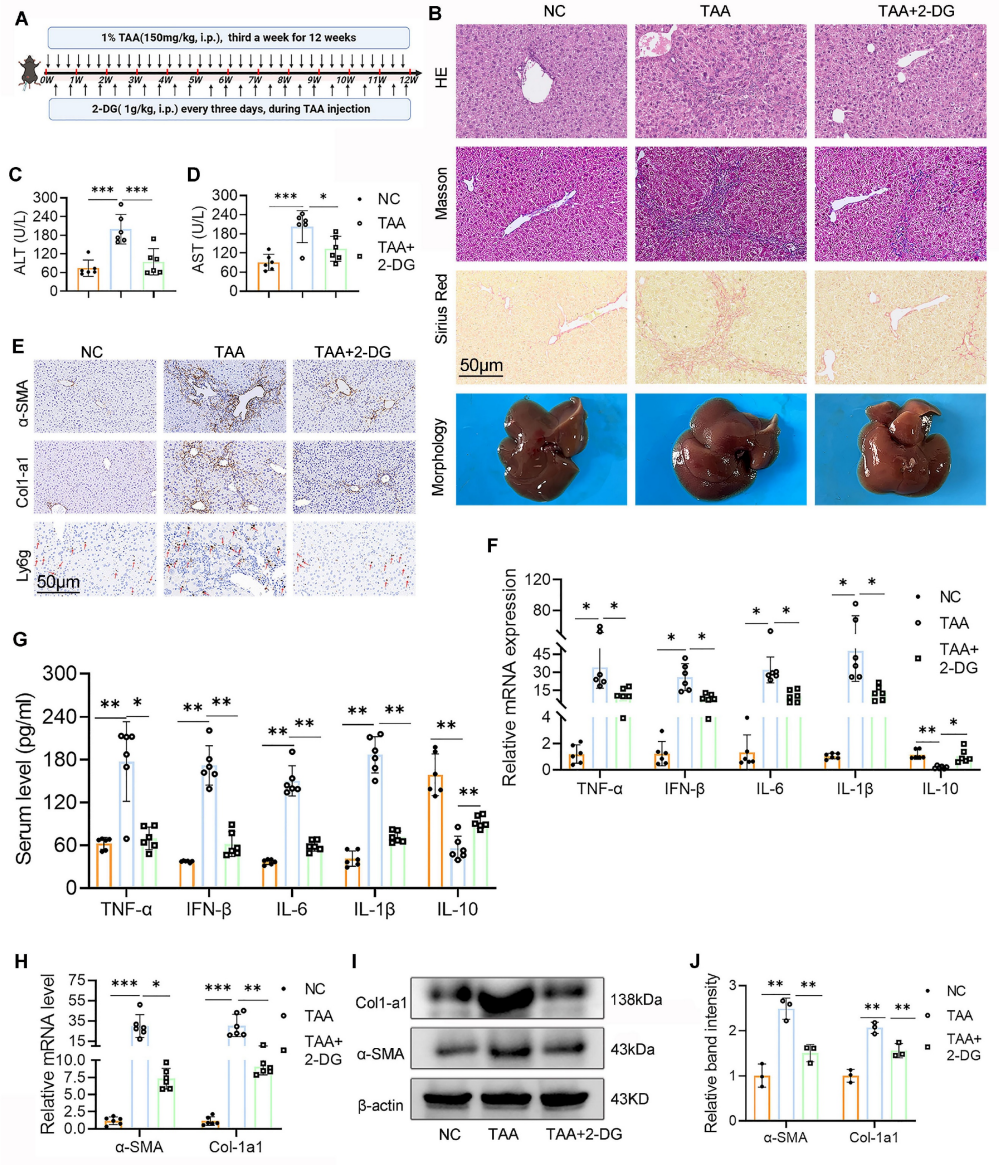
** 2-DG treatment ameliorates TAA-induced mice liver fibrosis (LF). (A)** Schematic diagram of liver fibrosis induction and 2-DG administration. **(B)** Representative images of liver morphology and their HE, Masson and Sirius red staining in NC, TAA and TAA+2-DG group mice. Scale bar = 50 μm. **(C-D)** Sreum ALT and AST levels across different group mice, n=6. **(E)** Representative IHC images of α-SMA, Col1-a1 and Ly6g in mice liver sections. Scale bar = 50μm. **(F-G)** Inflammatory cytokines including TNF-α, IFN-β, IL-6, IL-1β and IL-10 levels in mice serum and liver tissues were measured by ELISA and RT-qPCR, respectively, n=6. **(H)** mRNA expression of α-SMA and Col1-a1 in mice liver tissues, n=6. **(I-J)** Western blot and quantitative analysis of α-SMA and Col1-a1 in mice liver tissues. *p < 0.05, **p < 0.01, ***p < 0.001.

To examine the therapeutic potential of glycolysis inhibition, LF was induced in mice by repeated i.p. injections of TAA, with concurrent administration of the glycolysis inhibitor 2-DG throughout the treatment period (Fig. 2A). Histological analyses, including H&E, Masson's trichrome, and Sirius Red staining, showed marked hepatocyte swelling, inflammatory cell infiltration, extracellular matrix accumulation, and fibrotic changes in the livers of TAA-treated mice. In contrast, 2-DG-treated mice displayed substantial attenuation of these pathological features, along with reduced hepatic collagen deposition (Fig. 2B). Additionally, liver morphology in the 2-DG group was visibly improved compared with the TAA group. Serum biochemical analysis further revealed significantly lower ALT and AST levels in the 2-DG group (Figs. 2C, D).

Since chronic liver inflammation contributes to fibrosis^2^, we next evaluated hepatic inflammation and fibrotic status using ELISA, reverse transcription polymerase chain reaction (RT-PCR), and WB analysis of serum and liver tissue. The TAA group exhibited elevated mRNA and serum levels of proinflammatory cytokines, including TNF-α, IFN-β, IL-6, and IL-1β, all of which were significantly reduced following 2-DG treatment. Conversely, 2-DG administration led to an increase in the anti-inflammatory cytokine IL-10 (Figs. 2F-G). IHC staining showed fewer Ly6g-positive neutrophils in liver sections from 2-DG-treated mice compared with those from the TAA group (Fig. 2E). Moreover, mRNA and protein levels of fibrosis markers (α-SMA and Col-1a1) were markedly upregulated in the TAA group but significantly suppressed in the 2-DG group (Figs. 2E, H-J).

In summary, these results demonstrate that glycolysis inhibition by 2-DG effectively mitigates hepatic inflammation and fibrosis, thereby improving liver function in the TAA-induced LF model.

### Inhibition of glycolysis suppressed macrophage M1 polarization both *in vivo* and *in vitro*

**Figure 3:**
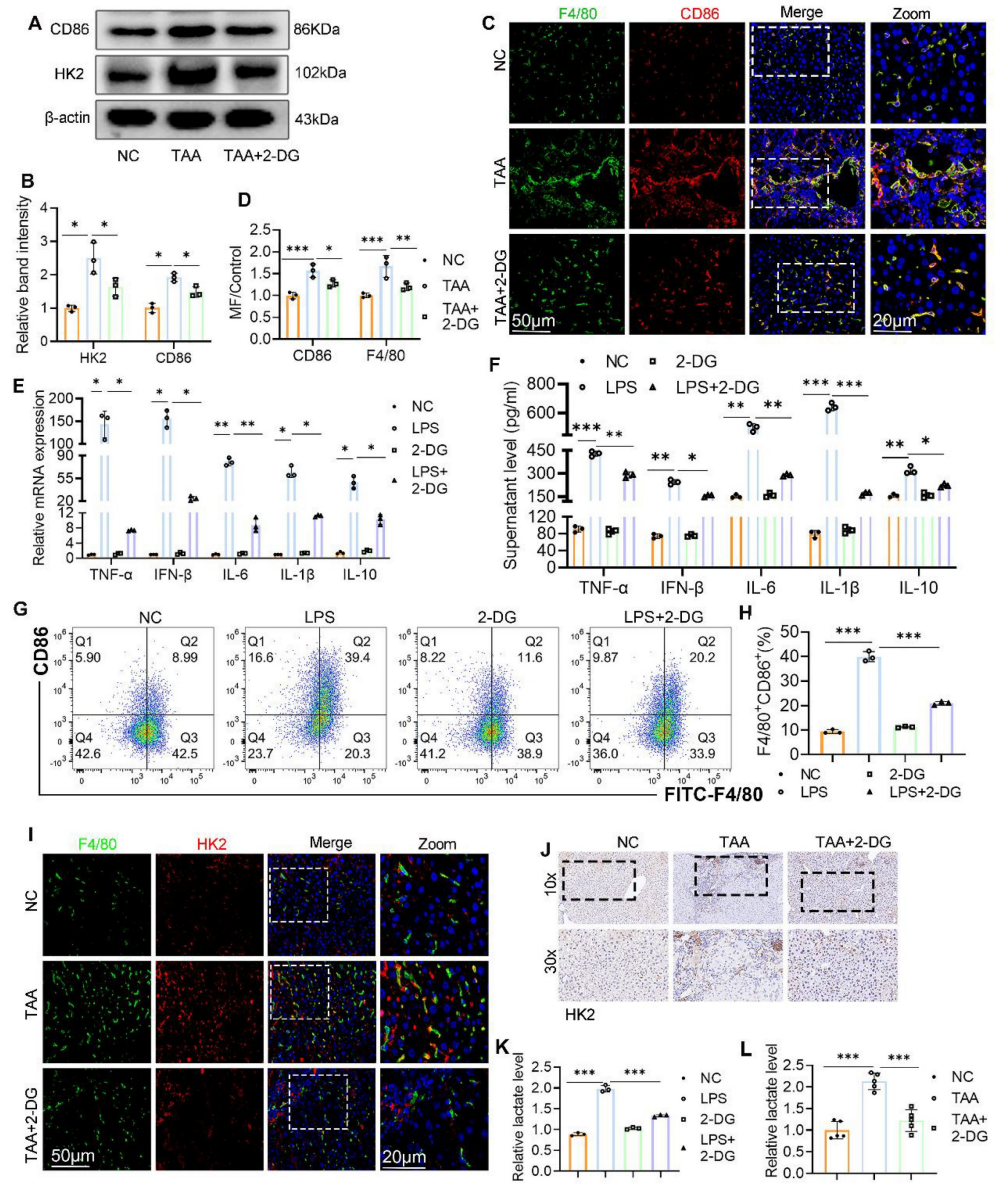
** Inhibition of glycolysis suppressed macrophage M1 polarization both *in vivo* and *in vitro*. (A-B)** Western blot and quantification of CD86 and HK2 in liver tissues from NC, TAA and TAA+2-DG group mice. **(C)** Double IF staining images of F4/80 (green) and CD86 (red) in mice liver sections from three groups. Scale bar = 50μm. **(D)** Relative quantification of fluorescence intensity of (C). MF: mean fluorescence intensity. **(E-F)** RT-qPCR and ELISA analysis of TNF-α, IFN-β, IL-6, IL-1β and IL-10 in cultured BMDMs after treated with LPS and 2-DG, n=6. **(G-H)** Flow cytometry analysis of macrophage polarization in LPS- and 2-DG-treated BMDMs. **(I)** Representative IF images of mice liver section which double-labeled F4/80 and HK2. Scale bars, 50 μm. **(J)** Representative IHC images of HK2 in mice liver. Magnification, 10x; zoom at 30x. **(K)** Relative lactate production in BMDMs among different groups. **(L)** Relative lactate level in mice liver tissues from NC, TAA and TAA+2-DG group, n=6. *p < 0.05, **p < 0.01, ***p < 0.001.

**Figure 4:**
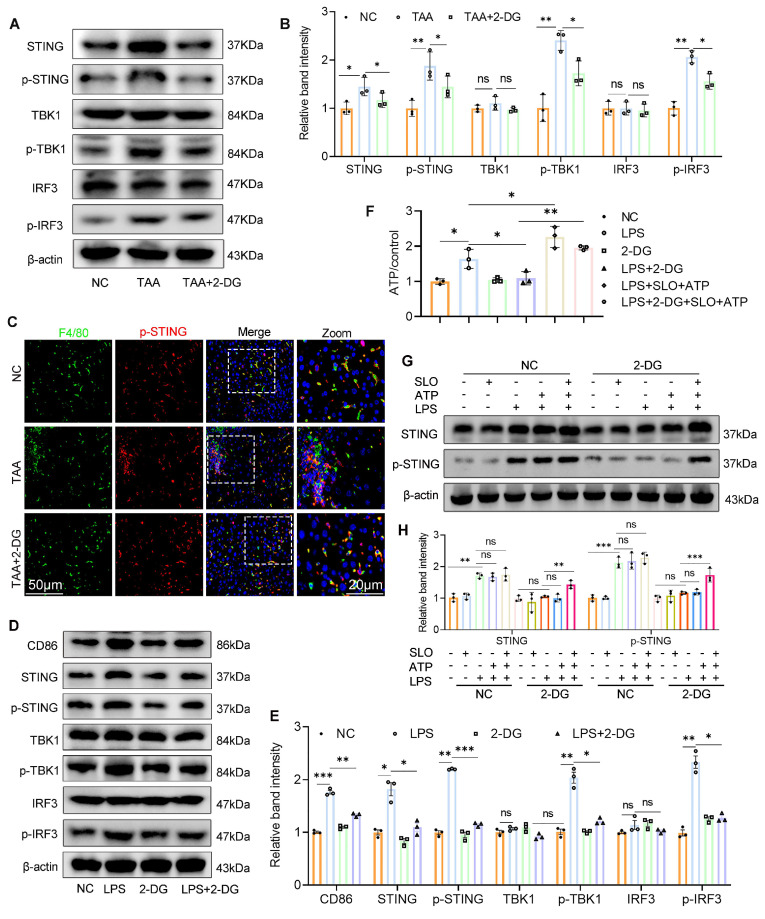
** Inhibition of glycolysis suppresses STING signaling activation by reducing glycolytic ATP production. (A-B)** Immunoblot analysis and corresponding quantification of STING, p-STING, IRF3, p-IRF3, TBK1 and p-TBK1 in mice liver tissues. **(C)** Double IF staining of F4/80 and p-STING in mice liver sections from different groups. Scale bar, 50 μm. **(D-E)** Western blot and quantification of CD86 and STING pathway proteins. *p < 0.05, **p < 0.01, ***p < 0.001. **(F)** Relative ATP content in cultured BMDMs. **(G-H)** WB analysis of STING and p-STING in BMDMs stimulated with LPS (100 ng/ml) in the presence of SLO and ATP for 12 hours. BMDMs were pretreated with 2-DG (5 mM) for 12 h and subsequently treated with LPS, SLO and ATP.

Chronic inflammatory stimuli reprogram hepatic glucose metabolism, promoting macrophage polarization toward the proinflammatory M1 phenotype and accelerating fibrosis progression^20^. To explore the effect of glycolysis inhibition on macrophage polarization, we first examined CD86 expression in liver tissue. WB analysis showed a marked reduction in CD86 levels following 2-DG treatment (Figs. 3A, B). IF staining further confirmed this finding, revealing a decreased number of F4/80^+^CD86^+^ macrophages in liver sections from 2-DG-treated mice compared with the TAA group (Figs. 3C-D). To validate these observations *in vitro*, BMDMs were stimulated with LPS in the presence or absence of 2-DG. RT-qPCR and ELISA analyses revealed that 2-DG significantly suppressed both mRNA and protein expression of inflammatory cytokines, including TNF-α, IFN-β, IL-6, IL-1β, and IL-10 (Figs. 3E, F). Flow cytometry and WB further demonstrated that 2-DG reduced M1 polarization: both the percentage of F4/80^+^CD86^+^ cells and CD86 protein levels were significantly lower in the 2-DG+LPS group than in the LPS group alone (Figs. 3G-H, Fig. 4D-E, respectively).

WB and IHC analyses also revealed that 2-DG attenuated the upregulation of HK2 in TAA-treated liver tissue (Figs. 3A, B, and J). In support of this, double IF staining showed a notable increase in F4/80^+^ cells and their HK2 intensity in the TAA group, both of which were substantially reduced following 2-DG administration (Fig. 3I). Because lactate is a key end product of glycolysis, we assessed lactate levels in liver tissue and BMDMs. Lactate concentrations were significantly reduced in the 2-DG-treated groups compared with both the TAA and LPS groups (Figs. 3K, L).

These findings indicate that enhanced glycolysis is a defining feature of fibrotic liver tissue and that glycolysis inhibition suppresses macrophage M1 polarization and the associated inflammatory response in both *in vivo* and *in vitro* models.

### Inhibition of glycolysis suppresses STING signaling activation by reducing glycolytic ATP production

Given the pivotal role of the STING pathway in innate immunity and macrophage-mediated hepatic inflammation^10^, ^21^ and emerging evidence suggesting that glycolysis can activate STING signaling^14^ we therefore investigated whether 2-DG exerts its anti-inflammatory effects via modulation of this pathway.

WB analysis revealed that both TAA exposure *in vivo* and LPS stimulation *in vitro* activated the STING signaling cascade, as shown by increased expression of STING, p-STING, phosphorylated TBK1 (p-TBK1), and phosphorylated IRF3 (p-IRF3), compared with the NC group (Figs. 4A-B and 4D-E). In contrast, 2-DG treatment markedly reduced the expression of these molecules in fibrotic liver tissue and LPS-stimulated BMDMs. IF staining further confirmed increased p-STING levels in liver macrophages after TAA administration, which were significantly diminished following 2-DG treatment (Fig. 4C). These results strongly support the role of glycolysis in driving STING pathway activation in macrophages.

As ATP production is closely linked to glycolytic activity^22^, we next measured ATP levels in activated BMDMs. LPS stimulation significantly increased intracellular ATP, whereas 2-DG treatment sharply reduced ATP levels (Fig. 4F). To determine whether ATP depletion underlies the suppression of STING signaling, we delivered exogenous ATP to 2-DG-treated BMDMs using streptolysin-O. Notably, intracellular ATP was restored, and STING pathway activation resumed, confirming that glycolytic ATP is essential for maintaining STING signaling (Figs. 4G-H).

In summary, our data demonstrate that inhibition of glycolysis suppresses STING signaling in activated macrophages by reducing glycolytic ATP production, thereby mitigating hepatic inflammation.

### Overexpression of HK2 in hepatic macrophages aggravates liver inflammation and fibrosis

To further investigate whether enhanced glycolysis in macrophages contributes to LF, we focused on HK2, a critical rate-limiting enzyme in the glycolytic pathway. HK2 was selectively overexpressed in hepatic macrophages via intrahepatic injection of AAV9 carrying an HK2-expressing vector under the control of the macrophage-specific F4/80 promoter (AAV9-F4/80-HK2). As a control, mice received an empty vector with the same macrophage-specific promoter (AAV9-F4/80-NC).

**Figure 5:**
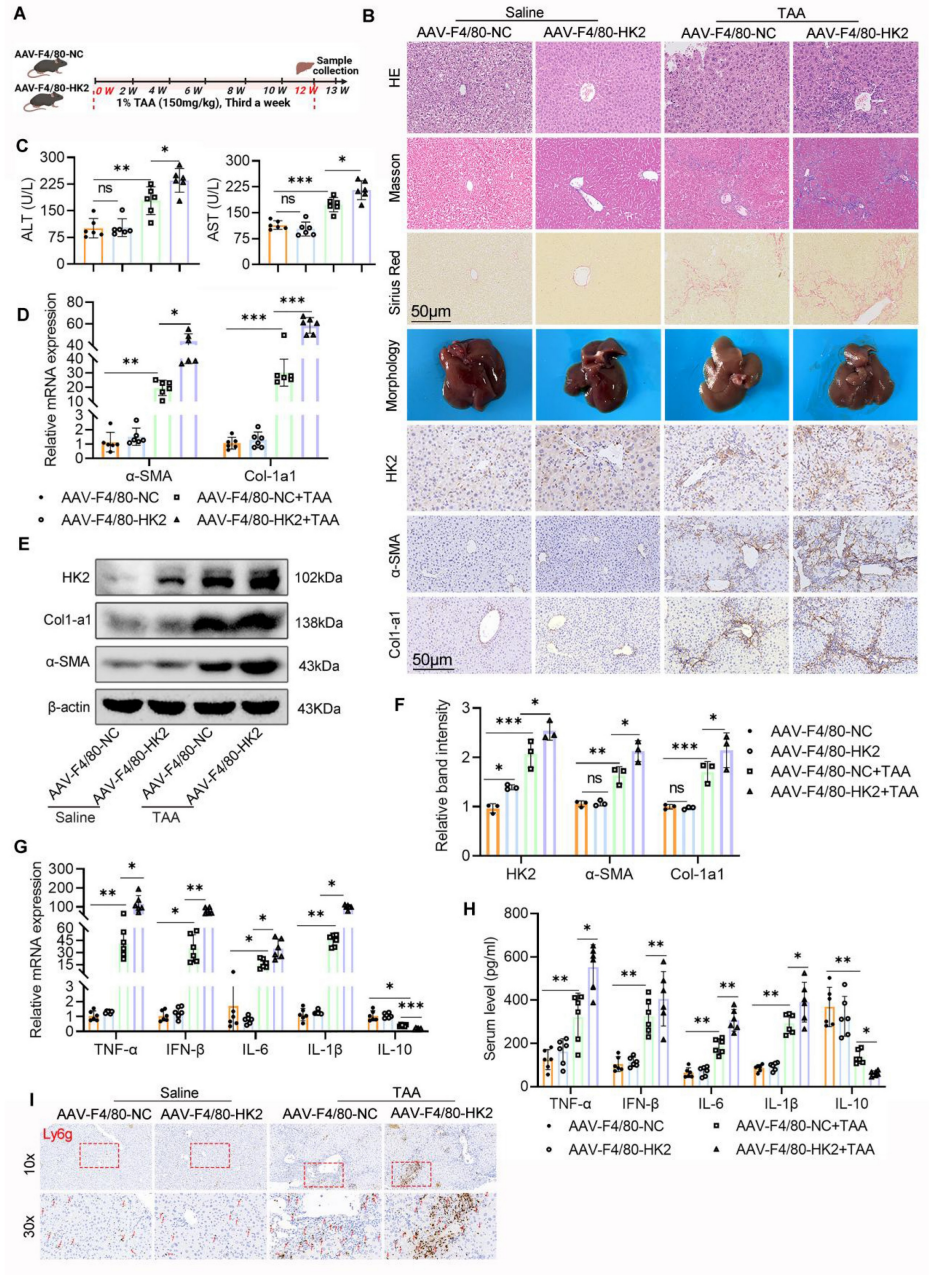
** Over-expression of HK2 in hepatic macrophages significantly aggravates mice liver inflammation and fibrosis. (A)** A schematic diagram depicting the liver fibrosis model induction in AAV-F4/80-NC and AAV-F4/80-HK2 mice. **(B)** Pathological staining (HE, Masson and Sirius red staining), IHC staining of HK2, α-SMA and Col1-a1 of liver sections in different group mice. Scale bar, 50 μm. **(C)** Serum level of ALT and AST were measured using ELISA, n=6. **(D)** mRNA expression of α-SMA and Col1-a1 in mice liver tissues, n=6. **(E-F)** Immunoblot and relative band intensity of HK2, α-SMA and Col1-a1 in mice liver tissues. **(G-H)** RT-qPCR and ELISA analysis of TNF-α, IFN-β, IL-6, IL-1β and IL-10 in the serum and liver tissues from various groups, respectively, n=6. **(I)** Representative IHC images of Ly6g in mice liver sections. *p < 0.05, **p < 0.01, ***p < 0.001.

**Figure 6:**
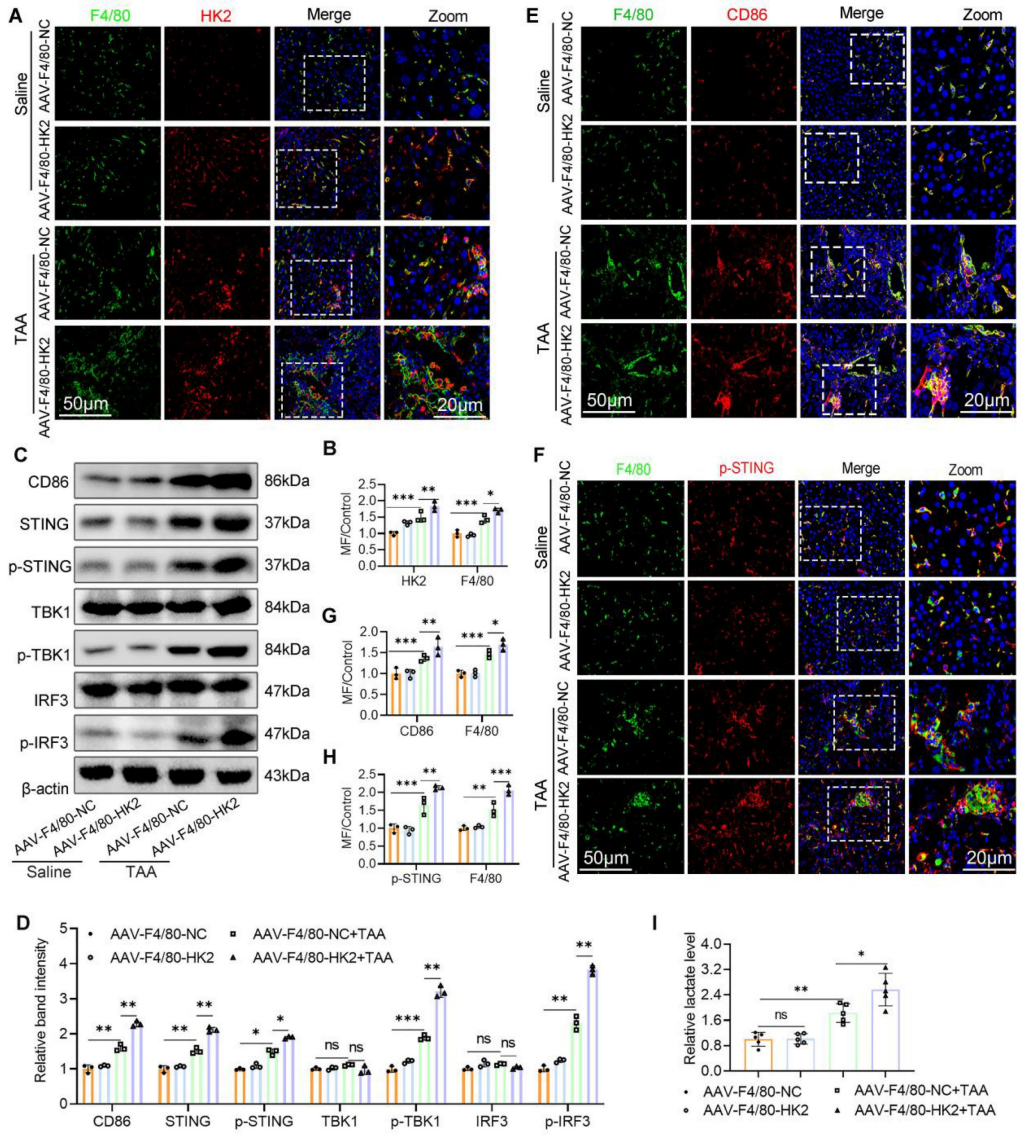
** Over-expression of macrophage HK2 augments STING pathway activation glycolysis and promotes, M1 polarization in the fibrotic liver. (A-B)** Double IF staining of F4/80 (green) and HK2 (red) in mice liver sections from different groups. Scale bar, 50 μm. **(C)** Protein expression of CD86 and STING, p-STING, IRF3, p-IRF3, TBK1 and p-TBK1 in mice liver tissues were detected using western blot. **(D)** Gray analysis of the western blot results in (C). **(E)** Analysis of M1 macrophage polarization using dual-IF staining of F4/80 and CD86 in mice liver from various groups. **(F)** Dual-IF staining of p-STING and F4/80 in mice liver sections. **(G-H)** Quantification analysis of IF staining results in (E) and (F), respectively. (I) Relative lactate production in mice liver tissues, n=6. *p < 0.05, **p < 0.01, ***p < 0.001.

Following viral delivery, mice injected with either AAV9-F4/80-HK2 or AAV9-F4/80-NC were subjected to the TAA-induced LF model (Fig. 5A). IHC, WB, and double IF staining confirmed significantly elevated HK2 expression in the livers of AAV9-F4/80-HK2 mice, particularly localized to macrophages, when compared with control mice (Fig. 5B, E-F and Figs. 6A-B). Although the HK2 overexpression alone did not affect liver morphology under basal conditions, the mice in the AAV9-F4/80-HK2 + TAA group displayed markedly aggravated fibrotic injury following TAA administration. This was characterized by extensive cytoplasmic vacuolization, pronounced lobular inflammatory infiltration, and increased collagen deposition, as demonstrated by H&E, Masson's trichrome, and Sirius Red staining (Fig. 5B). Livers from the AAV9-F4/80-HK2+TAA group also appeared rougher and stiffer compared with those from the AAV9-F4/80-NC+TAA group. Correspondingly, serum ALT and AST levels were significantly higher in the HK2-overexpressing group, indicating worsened liver function (Fig. 5C).

The profibrotic effects of HK2 overexpression were further validated by increased expression of Col1a1 and α-SMA in liver tissues from AAV9-F4/80-HK2 + TAA mice (Figs. 5B, D-F). Additionally, RT-PCR and ELISA analyses showed markedly higher levels of proinflammatory cytokines (TNF-α, IFN-β, IL-6, and IL-1β) in the HK2-overexpressing group compared with controls. Conversely, both mRNA and serum levels of the anti-inflammatory cytokine IL-10 were significantly reduced (Figs. 5G, H). IHC staining for Ly6g further revealed increased hepatic neutrophil infiltration in the AAV9-F4/80-HK2+TAA group (Fig. 5I).

Collectively, these results confirm that overexpression of HK2 in hepatic macrophages intensifies liver inflammation and accelerates fibrosis progression.

### Over-expression of MФ HK2 augments STING pathway activation, increases glycolysis, and promotes M1 polarization in fibrotic liver

Building on our previous findings that glycolysis inhibition attenuates LF, possibly through modulating M1 macrophage polarization and STING signaling, we next evaluated the consequences of enhanced macrophage glycolysis on STING pathway activity. WB analysis revealed that HK2 overexpression significantly upregulated the expression of CD86, STING, p-STING, p-TBK1, and p-IRF3 in liver tissues from the AAV9-F4/80-HK2+TAA group compared with the control group (Figs. 6C, D). IF staining further demonstrated that HK2 overexpression markedly increased both the number of M1 macrophages and the expression of p-STING within fibrotic liver sections (Figs. 6E-H). Additionally, hepatic lactate levels were notably elevated in the AAV9-F4/80-HK2+TAA group compared with the AAV9-F4/80-NC+TAA group (Fig. 6I).

### Myeloid STING deficiency alleviates TAA-induced mice liver inflammation and fibrosis

**Figure 7:**
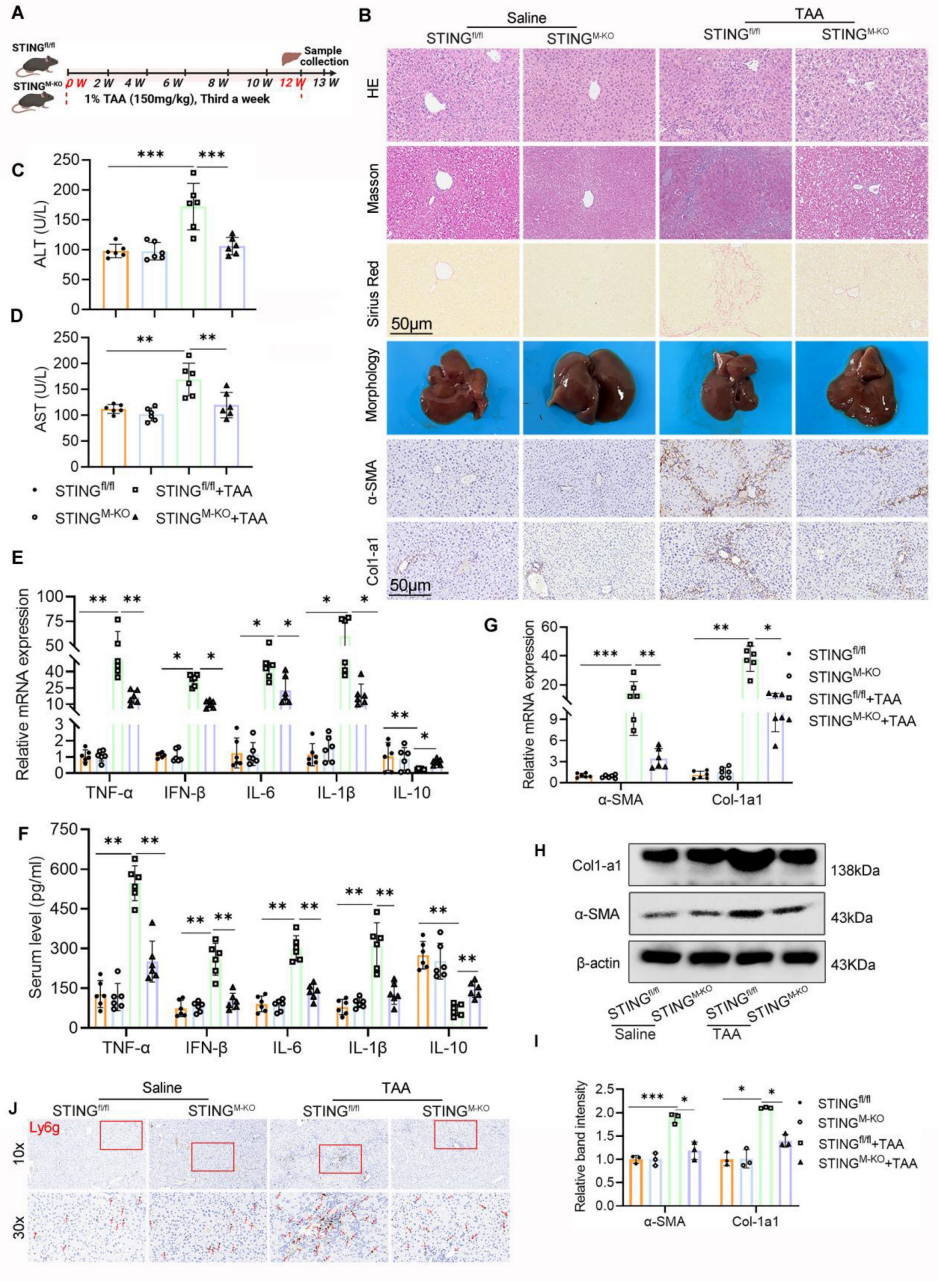
** Myeloid STING deficiency alleviates TAA-induced mice liver inflammation and fibrosis. (A)** A schematic diagram illustrating the liver fibrosis induction in STING^fl/fl^ and STING^M-KO^ mice. **(B)** Liver sections obtained from different group mice were used for pathological analysis (HE, MASSON and Sirius red staining) and IHC staining of α-SMA and Col1-a1. Scale bar = 50μm. **(C-D)** ELISA analysis of serum levels of ALT and AST, n=6. **(E-F)** mRNA and serum levels of Inflammatory cytokines including TNF-α, IFN-β, IL-6, IL-1β and IL-10 were determined using RT-qPCR and ELISA, respectively, n=6. **(G-I)** mRNA and protein expression of Col1-a1 and α-SMA in mice liver tissues among different groups, n=6. **(J)** representative IHC images of Ly6g in mice liver sections in various groups. *p < 0.05, **p < 0.01, ***p < 0.001.

The above findings established a critical role for STING in LF and macrophage glycolysis activation. To further delineate STING's function in this context, we generated myeloid cell-specific STING knockout mice (STING^M-KO^) by crossing STING^fl/fl^ mice with LyZ2-Cre+ mice. Successful recombination was confirmed by tail genotyping (Figs. S1A-B). WB, IHC, and double IF staining confirmed a marked reduction in STING expression specifically within macrophages in STING^M-KO^ mice (Figs. S1C-E). STING^M-KO^ and STING^fl/fl^ mice were then subjected to TAA i.p. injections to induce LF (Fig. 7A).

Histopathological and IHC analyses revealed markedly less hepatic inflammation and ECM accumulation in STING^M-KO^ mice compared with TAA-treated STING^fl/fl^ controls. There was a notable decrease in α-SMA and Col1a1 expression in the STING-deficient livers (Fig. 7B). Despite TAA treatment, the livers of STING^M-KO^ mice appeared smoother and softer than those of STING^fl/fl^ counterparts. Biochemically, serum ALT and AST levels were significantly lower in the TAA-treated STING^M-KO^ group, indicating improved liver function (Figs. 7C, D). Further evaluation of α-SMA and Col1a1 levels by IHC, WB, and RT-PCR, along with Ly6g IHC and measurements of inflammatory cytokines (TNF-α, IFN-β, IL-6, IL-1β, and IL-10) via RT-PCR and ELISA, showed that deletion of STING in macrophages substantially reduced TAA-induced profibrotic and proinflammatory responses (Figs. 7B, E-J).

Taken together, these data demonstrate that myeloid-specific STING deletion significantly mitigates liver inflammation and fibrosis.

### Myeloid STING deficiency suppresses glycolysis and M1 polarization in macrophage

**Figure 8:**
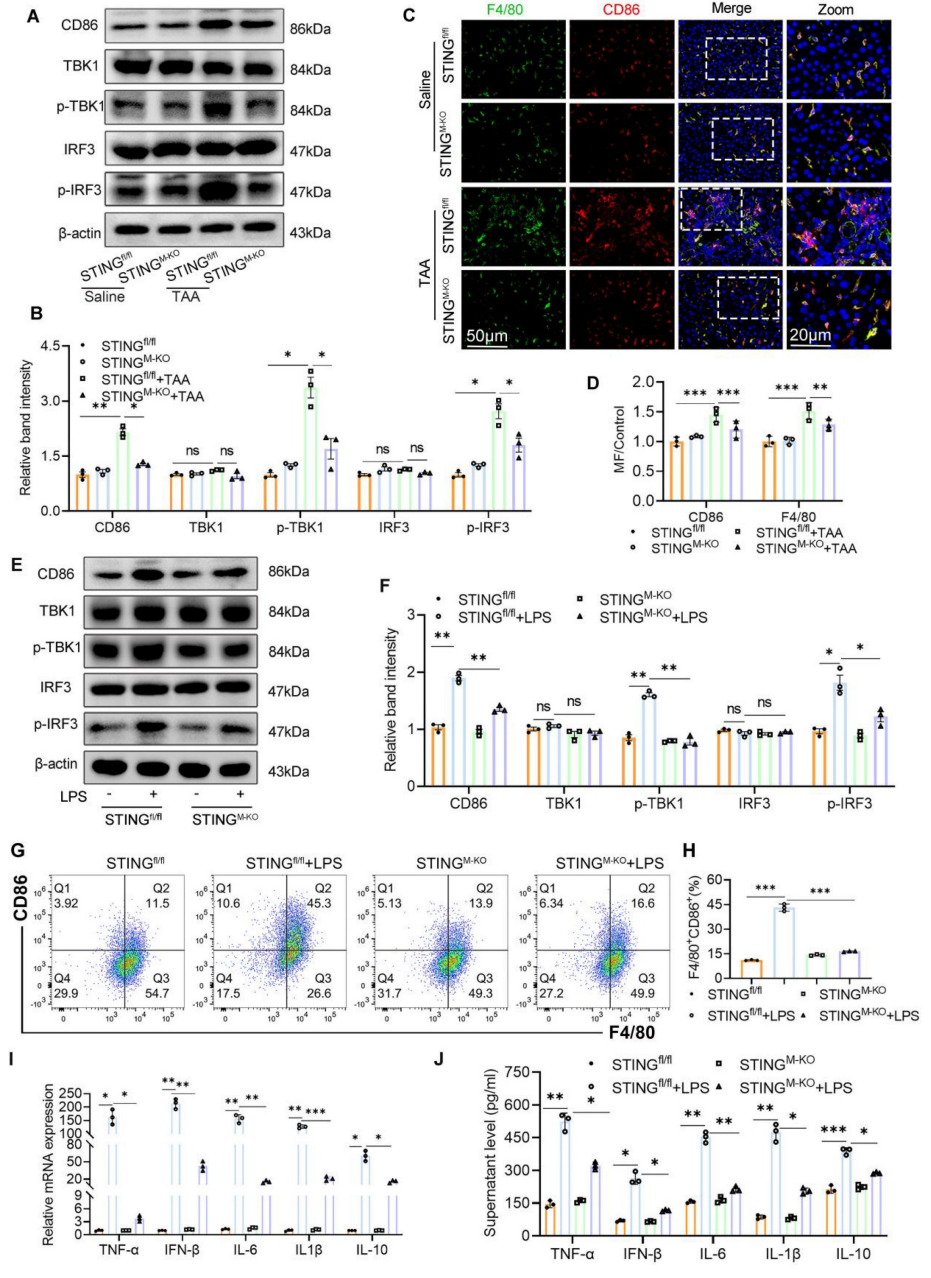
** Myeloid STING deficiency suppress glycolysis and M1 polarization in macrophage. (A)** Protein expression of CD86, TBK1, p-TBK1, IRF3 and p-IRF3 in liver tissues were determined by western blot. **(B)** Gray scale analysis of the western blot results in (A). **(C-D)** Dual-IF staining of F4/80 (green) and CD86 to assess the macrophage polarization in mice liver sections to assess macrophage polarization in mice liver among different groups. **(E-F)** Western blot and quantification analysis of CD86, TBK1, p-TBK1, IRF3 and p-IRF3 in BMDMs isolated from STING^fl/fl^ and STING^M-KO^ mice that treated with or without LPS. **(G-H)** Effect of STING knockout on the polarization of cultured BMDMs was determined by flow cytometry. **(I-J)** mRNA and supernatant levels of TNF-α, IFN-β, IL-6, IL-1β and IL-10 in cultured BMDMs, respectively. *p < 0.05, **p < 0.01, ***p < 0.001.

To investigate the mechanistic impact of STING deletion on macrophage phenotype and metabolic status, we assessed macrophage polarization and the expression of downstream STING signaling components. STING knockout markedly reduced the TAA-induced increase in hepatic F4/80^+^CD86^+^ macrophages and lowered the expression of CD86, p-TBK1, and p-IRF3 (Figs. 8A-D). In parallel, BMDMs derived from STING^fl/fl^ and STING^M-KO^ mice were stimulated with or without LPS. WB revealed significantly lower levels of CD86, p-TBK1, and p-IRF3 in LPS-stimulated STING^M-KO^ BMDMs compared with STING^fl/fl^ controls (Figs. 8E-F). Flow cytometry, qPCR, and ELISA further confirmed that STING deficiency reduced the proportion of M1 macrophages and lowered the expression of inflammatory cytokines, including TNF-α, IFN-β, IL-6, IL-1β, and IL-10 (Figs. 8G-J).

**Figure 9:**
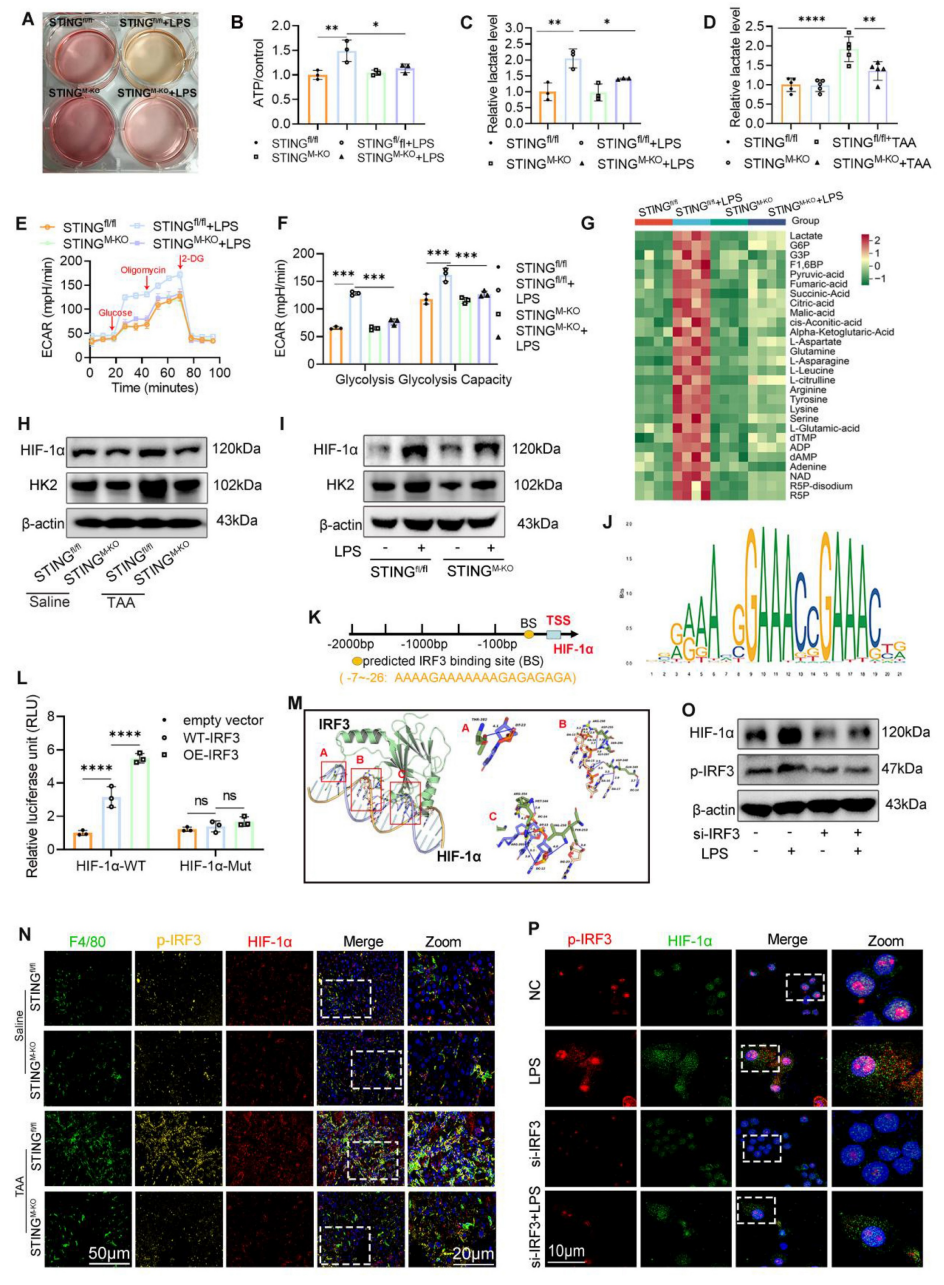
** HIF-1α regulated by IRF3 was involved in the activation of glycolysis and STING signaling. (A)** Representative image of the cell culture medium change. **(B-C)** Relative ATP and lactate production in BMDMs isolated from STING^fl/fl^ and STING^M-KO^ mice that treated with or without LPS, respectively. **(D)** Relative lactate level in mice liver tissues from different groups, n=6, respectively. **(E-F)** ECAR of BMDMs was detected by seahorse analyzer. **(G)** Glycolytic metabolites analysis of BMDMs isolated from STING^fl/fl^ and STING^M-KO^ mice that treated with or without LPS. **(H-I)** HK2 and HIF-1α protein levels in liver tissues and in cultured BMDMs, respectively. **(J-K)** Conserved motifs of IRF3 and schematic illustration of predicted IRF3 binding sites in the HIF-1α promoter region. **(L)** Relative luciferase activity in BMDMs determined by dual-luciferase assay. **(M)** The optimal binding model of the HIF-1α promoter DNA sequence with the IRF3 protein. **(N)** Immunofluorescence co-staining of F4/80 (green), HIF-1α (red) and p-IRF3 (yellow) in mice liver sections. **(O)** Effect of silence IRF3 on the protein expression of HIF-1α and p-IRF3 in BMDMs. **(P)** Representative IF staining of co-localization of HIF-1α and p-IRF3 in BMDMs with different treatment. *p < 0.05, **p < 0.01, ***p < 0.001.

Interestingly, during LPS stimulation, the culture medium of STING^M-KO^ BMDMs changed color more slowly than that of STING^fl/fl^ BMDMs, suggesting reduced acid production and altered metabolic activity (Fig. 9A). We hypothesized that STING deficiency impairs aerobic glycolysis. Indeed, under basal conditions, there were no significant differences in cellular ATP content or lactate production between STING^fl/fl^ and STING^M-KO^ BMDMs. However, following LPS exposure, ATP and lactate levels were significantly elevated in STING^fl/fl^ BMDMs but were markedly reduced in STING^M-KO^ BMDMs (Figs. 9B, C).

Seahorse metabolic analysis showed comparable basal ECAR between groups, but upon glucose and oligomycin addition during LPS stimulation, STING^M-KO^ BMDMs displayed significantly reduced ECAR, indicating impaired glycolytic capacity (Figs. 9E-F). Correspondingly, STING knockout reduced the TAA-induced increase in hepatic lactate levels and HK2 expression (Fig. 9D, 9H). IF staining further confirmed decreased HK2 expression in hepatic macrophages from the STING-deficient group following TAA exposure (Fig. S2A).

To corroborate these findings, we performed central carbon metabolome profiling of BMDMs from STING^fl/fl^ and STING^M-KO^ mice, with or without LPS stimulation. Heatmap analysis revealed that several metabolites elevated by LPS were significantly suppressed in STING-deficient macrophages, even in the presence of LPS (Fig. 9G). Notably, the altered metabolites were closely linked to the glycolytic pathway and tricarboxylic acid (TCA) cycle, including dTMP, NAD, succinate, fumarate, pyruvate, and lactate.

Collectively, these results confirm that STING is a critical driver of aerobic glycolysis and lactate production in macrophages, thereby promoting M1 polarization and inflammatory activation during LF.

### IRF3-regulated HIF-1α contributes to the activation of glycolysis and STING signaling

The above findings suggest that glycolysis enhances STING signaling activation, whereas STING deficiency suppresses glycolytic activity. These observations imply the presence of a positive feedback loop between glycolysis and STING signaling. HIF-1α is a well-established transcription factor that drives glycolysis and promotes ATP production by upregulating key glycolytic enzymes, including HK2^23^. Consistently, we observed that both TAA and LPS treatments increased HIF-1α and HK2 expression in liver tissues and BMDMs, respectively, whereas STING deficiency significantly reduced these inductions (Figs. 9H-I and Fig. S2B-C). Since STING activates type I IFN gene expression via phosphorylation and nuclear translocation of IRF3^24^, we hypothesized that p-IRF3 transcriptionally regulates HIF-1α expression. Using Animal TFDB (https://guolab.wchscu.cn/AnimalTFDB4/#/), we predicted potential p-IRF3 binding sites in the HIF-1α-promoter region (Figs. 9J-K). Based on binding affinity scores, we selected the highest-scoring binding site and constructed both WT (pGL3-HIF-1α-WT) and mutated (pGL3-HIF-1α-Mut; 5′→3′: “TCTCTCTCTTTTTTTCTTTT” mutated to “AGAGAGAGAAAAAAAGAAT”) promoter-reporter plasmids. These were cotransfected into BMDMs with either an empty vector (pEnCMV-3xflag-NC) or an IRF3 overexpression plasmid (pEnCMV-3xflag-IRF3, OE-IRF3).

Dual-luciferase assays showed significantly higher luciferase activity in the WT-IRF3+HIF-1α-WT group compared with vector control, and this activity was further enhanced by OE-IRF3. In contrast, the mutated promoter markedly suppressed luciferase activity (Fig. 9L). Protein-DNA docking analysis also revealed a strong binding conformation between IRF3 protein and the *HIF-1α* promoter (Fig. 9M). IF staining of liver sections showed that TAA increased both p-IRF3 and HIF-1α expression in hepatic macrophages, which was attenuated in STING-deficient mice (Fig. 9N).

To directly assess the regulatory effect of IRF3 on HIF-1α, we silenced IRF3 expression in BMDMs using siRNA. WB analysis confirmed the knockdown efficiency, with si-1 demonstrating the greatest reduction (Figs. S3A-B). Notably, IRF3 knockdown significantly reduced HIF-1α expression even after LPS stimulation (Fig. 9O, Fig. S3C). Dual IF staining further confirmed that IRF3 silencing inhibited both IRF3 nuclear translocation and LPS-induced HIF-1α upregulation (Fig. 9P).

Together, these data demonstrate that IRF3 directly regulates HIF-1α transcription and that STING signaling promotes glycolysis through the IRF3-HIF-1α axis, establishing a feedforward loop between inflammatory signaling and metabolic reprogramming.

## Discussion

In this study, we demonstrated that glycolysis is significantly upregulated in both patients with cirrhosis and mouse models of LF, and that pharmacological inhibition of glycolysis markedly attenuates fibrosis progression. Mechanistically, we showed that glycolysis-derived ATP enhances activation of the STING/TBK1/IRF3 signaling pathway, triggering a type I IFN response and promoting M1 macrophage polarization. Moreover, STING activation upregulated HIF-1α, a key regulator of glycolysis, thereby forming a positive feedback loop that amplifies both metabolic and inflammatory responses. Collectively, these findings suggest that targeting dysregulated glucose metabolism in macrophages may offer a promising therapeutic strategy for managing cirrhosis.

Chronic injury and unresolved inflammation are central to the initiation and progression of LF^25^, ^26^. Macrophages, as central effectors of innate immunity, demonstrate plasticity by shifting between M1 (proinflammatory) and M2 (anti-inflammatory) phenotypes in response to environmental cues. M1 macrophages contribute to fibrosis by activating HSCs, recruiting inflammatory cells, and promoting ECM deposition^27^. Importantly, M1 polarization is accompanied by metabolic reprogramming from oxidative phosphorylation to glycolysis, enabling the production of ATP and biosynthetic intermediates required for inflammatory responses^28^**.** In addition to meeting metabolic demands, glycolysis generates signaling metabolites—such as lactate and succinate—that act as secondary messengers to promote inflammation. Lactate stabilizes HIF-1α, while succinate accumulation activates the NLRP3 inflammasome and promotes IL-1β secretion, amplifying immune responses^29^,^30^, ^31^.

In line with these findings, our study showed that 2-DG effectively inhibited LPS-induced M1 polarization of BMDMs, decreased lactate production, and reduced inflammatory cytokine release. Furthermore, *in vivo* administration of 2-DG mitigated TAA-induced hepatic inflammation and fibrosis, evidenced by lower serum ALT/AST levels, reduced Ly6g^+^ neutrophil infiltration, and decreased numbers of M1 macrophages in the liver. These findings reinforce the concept that glycolysis plays a central role in driving macrophage-mediated inflammatory responses in fibrotic liver disease.

Although HK2 is the initial rate-limiting enzyme in the glycolytic pathway, most previous studies on glycolytic enzymes in liver pathology have focused on pyruvate kinase M2 or GLUT1, often overlooking the role of HK2. In our study, however, HK2 emerged as a key regulator of macrophage glycolysis during liver fibrosis. Its overexpression in macrophages enhanced M1 polarization and increased neutrophil infiltration within the fibrotic liver, leading to exacerbated hepatic dysfunction. These results suggest that selectively targeting HK2-dependent glycolysis may offer a novel therapeutic approach to mitigate macrophage-driven inflammation and tissue damage in cirrhosis.

Moreover, our study reveals a critical mechanistic link between glycolytic metabolism and innate immune activation. We demonstrate that ATP produced via glycolysis activates the STING/TBK1/IRF3 signaling axis, initiating type I interferon responses and reinforcing M1 macrophage polarization, consistent with recent findings in dendritic cells^14^. Notably, STING exhibits context-dependent functions in liver pathology. While its inhibition mitigates inflammation and lipid accumulation in lean NAFLD^32^, STING activation has been shown to suppress HBV replication and enhance antitumor immunity^33^, ^34^. However, most of these studies have focused on STING's antiviral and immunomodulatory properties, with limited attention to its metabolic roles. Our findings extend this understanding by showing that 2-DG suppresses, whereas HK2 overexpression enhances, STING/TBK1/IRF3 activation. Interestingly, STING deficiency in macrophages reduced glycolytic flux, ATP generation, and inflammatory activation, suggesting a bidirectional regulatory loop between metabolism and immune signaling.

IRF3, a key downstream effector of STING, is increasingly recognized for its metabolic functions. It regulates adipocyte differentiation^35^, and its activity is elevated in patients with NAFLD. Hepatocyte-specific IRF3 knockdown improves insulin sensitivity and glycemic control in HFD-fed mice^36^. In addition, IRF3 drives hypoxia-induced renal fibrosis via upregulation of PFKFB3-dependent glycolysis^37^. However, its role in hepatic glycolytic regulation and macrophage polarization remains insufficiently characterized. HIF-1α is widely acknowledged as a central regulator of glycolysis^23^. Our experiments revealed that IRF3 directly binds to the HIF-1α promoter, promoting its transcription, while IRF3 silencing downregulates HIF-1α even in the presence of inflammatory stimulation. Since IRF3 activation is phosphorylation-dependent, our dual IF analysis showing coexpression and nuclear colocalization of p-IRF3 and HIF-1α in hepatic macrophages—both *in vivo* and *in vitro*—offers mechanistic insight into how STING signaling influences macrophage glycolytic reprogramming.

Nevertheless, this study has limitations. While we identified macrophage glycolysis as a critical driver of LF, other glycolytic cell types, such as hepatocytes and HSCs, may also contribute to fibrogenesis. Although the AAV9 vector driven by the F4/80 promoter targets macrophages, potential transgene expression in other F4/80^+^ myeloid subsets cannot be excluded, possibly contributing to the observed effects. Furthermore, although STING deficiency led to decreased HIF-1α expression, further investigation is needed to establish whether HIF-1α is an indispensable downstream mediator of STING signaling in hepatic fibrosis.

## Conclusion

Our study demonstrates that TAA-induced LF is fueled by enhanced glycolysis in hepatic macrophages, primarily driven by HK2 upregulation. ATP generated through glycolysis activates the STING/TBK1/IRF3 pathway, promoting inflammation and upregulating HIF-1α, thereby establishing a positive feedback loop that intensifies glycolysis and inflammatory signaling. These findings offer novel mechanistic insight into how metabolic reprogramming amplifies innate immune responses and provide a compelling rationale for targeting glycolysis-STING signaling as a therapeutic strategy for LF and liver cirrhosis.

## Supplementary Material

Supplementary figures and tables.
