# Supplementary material for: Glycolysis drives STING signaling to promote M1-macrophage polarization and aggravate liver fibrosis
Source: Int J Biol Sci. 2025 Oct 1;21(14):6411–29. doi: 10.7150/ijbs.115073 (PMC12594590; doi:10.7150/ijbs.115073)
Supplement: Supplementary file 1 — Supplementary figures and tables. [file ijbsv21p6411s1.pdf]

## Supplementary Figure

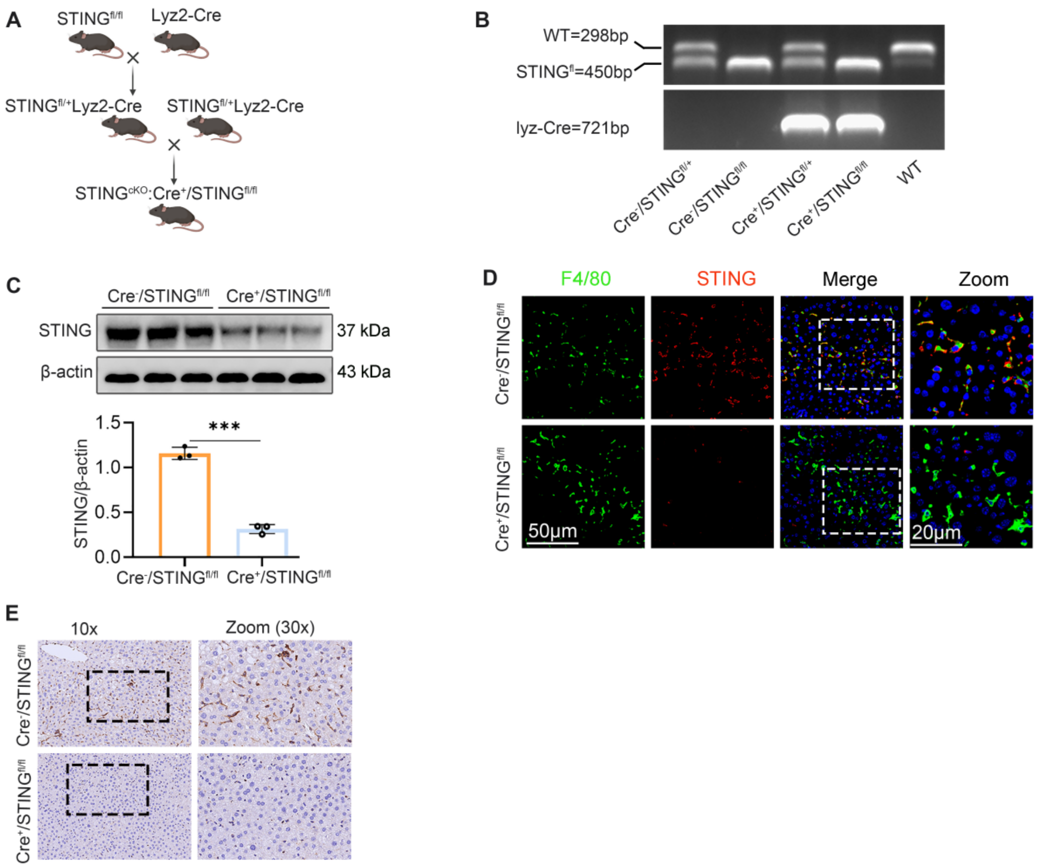


**Supplementary Figure 1. Identification of myeloid-specific STING knockout (STING$^{M\text{-}KO}$) mice**

(A) Schematic diagram for generation of conditional myeloid knockout of STING (STING$^{M\text{-}KO}$) by crossing STING$^{fl/fl}$ mice with Lyz2-Cre mice.

(B) Representative PCR image of tail genotyping.

(C) Western blot and gray scale analysis of STING protein level in the liver tissues among different group.

(D) Dual IF staining of STING and F4/80 to assess the STING expression in mice liver macrophages. Scale bar, 50 μm.

**(E)** IHC staining of hepatic STING. Magnification: 10x; zoom at 30x. *$p < 0.05$.

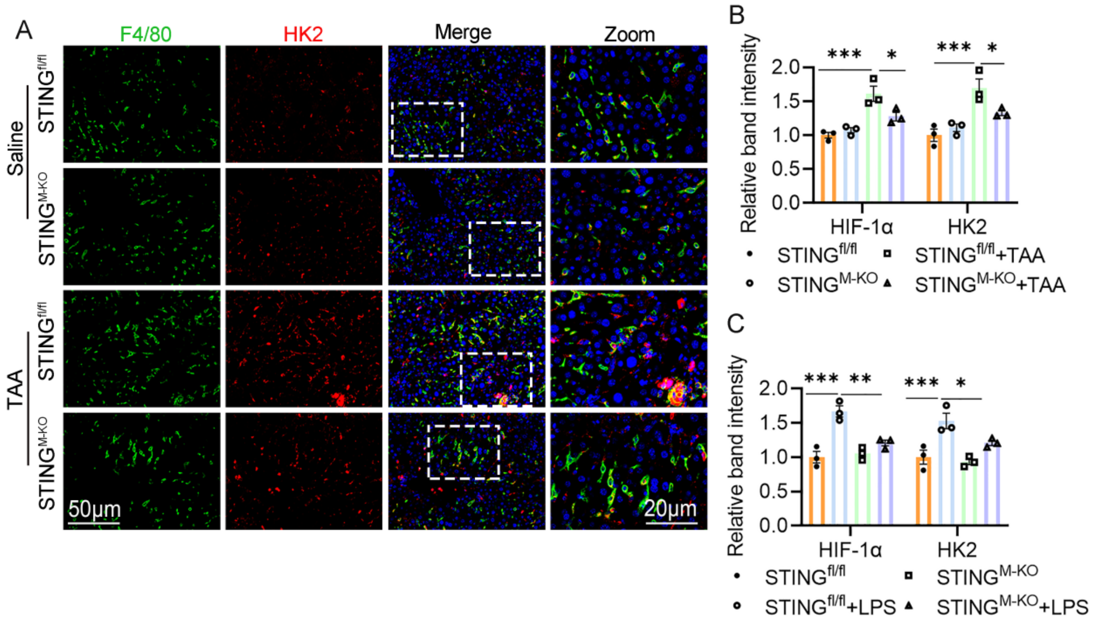


**Supplementary Figure 2. HK2 and HIF-1α expression in response to STING deletion**

(A) Double IF staining of HK2 and F4/80 in mice liver sections from $STING^{fl/fl}$, $STING^{M\text{-}KO}$, $STING^{fl/fl}$+TAA and $STING^{M\text{-}KO}$+TAA groups. Scale bar, 50 μm.

(B-C) Quantification analysis of the western blot results of HIF-1α and HK2 both in liver tissues and in BMDMs, respectively. $^*p < 0.05$, $^{**}p < 0.01$, $^{***}p < 0.001$.

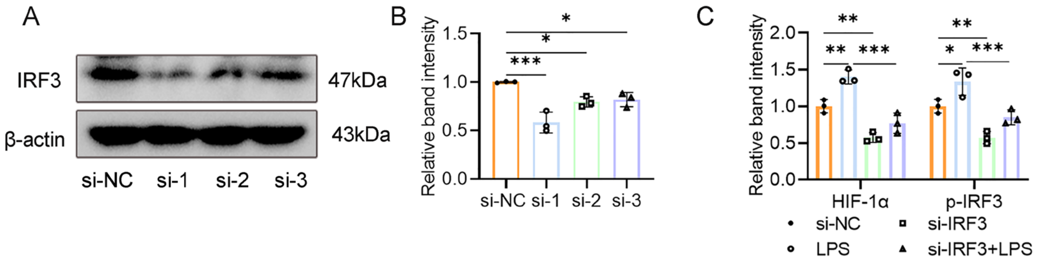


**Supplementary Figure 3. Analysis the effect of IRF3 silencing on HIF-1α**

(A-B) Representative western blot and semi-quantification of IRF3 in BMDMs after transfected with siRNA targeting IRF3 or negative control.

(B) Semi-quantification of protein levels in Fig.9(O). $*p < 0.05$, $**p < 0.01$, $***p < 0.001$.

**Supplementary Tables**

**Supplementary Table 1. All primary and secondary antibodies used for WB, IF and IHC**

| Source | Antibody | Dilutions | Company | Item No. |
|---|---|---|---|---|
| Mouse | β-actin | 1:10000 | Proteintech | 66009-1-Ig |
| Rabbit | p-IRF3 | 1:1000 | Abclonal | AP0623 |
| Rabbit | IRF3 | 1:1000 | Abclonal | A24864 |
| Rabbit | p-TBK1 | 1:2000 | Abclonal | AP1026 |
| Rabbit | TBK1 | 1:2000 | Abclonal | A3458 |
| Rabbit | STING | 1:1000 | CST | 13647S |
| Rabbit | p-STING | 1:2000 | Immunoway | YP1518 |
| Rabbit | HIF-1α | 1:1000 | Immunoway | YT2133 |
| Mouse | HK2 | 1:1000 | Proteintech | 66974-1-Ig |
| Rabbit | HK2 | 1:100 | Zenbio | 24552 |
| Rabbit | PKM2 | 1:2000 | CST | 4053 |
| Rabbit | PFKP | 1:1000 | Proteintech | 13389-1-AP |
| Rabbit | α-SMA | 1:1000 | Abcam | ab124964 |
| Rabbit | Col1-a1 | 1:2000 | Absin | abs118788 |
| Rabbit | CD86 | 1:1000 | CST | 19589 |
| Rabbit | CD68 | 1:200 | Abcam | ab283654 |
| Rabbit | F4/80 | 1:300 | Abcam | ab300421 |
| Rabbit | Ly6g | 1:100 | CST | #87048 |
| HRP-conjugated Goat anti-Rabbit IgG (H+L) | | 1:5000 | Abclonal | AS014 |
| HRP-conjugated Goat anti-Mouse IgG (H+L) | | 1:5000 | Abclonal | AS003 |
| Goat anti-Rabbit IgG H&L (Alexa Fluor® 488) | | 1:500 | Abcam | Ab150077 |
| Donkey anti-Rabbit IgG H&L | | 1:500 | Abcam | ab150075 |

| | | | |
|---|---|---|---|
| (Alexa Fluor® 647) | | | |
| Goat Anti-Rabbit IgG H&L (Alexa Fluor® 594) | 1:500 | Abcam | ab150080 |

**Supplementary Table 2. All primer sequences used in RT-qPCR**

| Gene | Forward | Reverse |
|---|---|---|
| α-SMA | CCAGCTATGTGTGAAGAGGAAGA | TTGGTGATGATGCCGTGTTCTAT |
| Col-1a1 | CCCGAGGTATGCTTGATCTGTAT | TCCCTCGACTCCTACATCTTCTG |
| β-actin | GGCTGTATTCCCCTCCATCG | CCAGTTGGTAACAATGCCATGT |
| TNF-α | CCCTCACACTCAGATCATCTTCT | GCTACGACGTGGGCTACAG |
| IFN-β | CAGCTCCAAGAAAGGACGAAC | GGCAGTGTAACTCTTCTGCAT |
| IL6 | CCAAGAGGTGAGTGCTTCCC | CTGTTGTTCAGACTCTCTCCCT |
| IL-1β | GCAACTGTTCCTGAACTCAACT | ATCTTTTGGGGTCCGTCAACT |
| IL-10 | GCTGGACAACATACTGCTAACC | ATTTCCGATAAGGCTTGGCAA |

**Supplementary Table 3. ELISA kits (Elabscience, China) used to quantify the levels of inflammatory cytokines**

| **Inflammatory cytokines** | **Item No.** |
|---|---|
| Mouse TNF-α | E-EL-M3063 |
| Mouse IFN-β | E-EL-M0033 |
| Mouse IL-6 | E-EL-M0044 |
| Mouse IL-1β | E-EL-M0037 |
| Mouse IL-10 | E-EL-M0046 |
